# North Carolina

**Published:** 1897-03

**Authors:** 


*North Carolina.* An Act which proposes to regulate the practice of veterinary medicine and surgery in North Carolina requires examination before the State Board of Veterinary Medical Examiners, consisting of five members elected by the North Carolina Veterinary Medical Association. It provides that the board shall meet at least once a year, at such place as the Association may decide upon. Two members of the board may grant a temporary certificate to practice, which shall be in force only until the next regular meeting of the board, but in no case shall such temporary certificate be granted to any person who has been an unsuccessful applicant for a certificate before the board. The fee is to be ten dollars before issuing a certificate and five dollars before issuing a temporary certificate. The board shall have authority to adopt such by-laws and regulations as may be necessary, and shall fill all vacancies in said board. Members of the board shall receive compensation for their services, not to exceed four dollars per day and travelling expenses, the same to be paid from fees in the hands of the secretary. The board shall have power to rescind any certificate granted by it, upon satisfactory proof that the person thus licensed has been guilty of grossly immoral conduct. All persons having practised veterinary medicine and surgery exclusively as a profession for five years previous to the passage of this Act shall be allowed to practise after making affidavit and registering their names on or before January 1, 1898. This Act does not prohibit any member of the medical profession from prescribing for domestic animals in cases of emergency and collecting a fee therefor, nor to prevent gratuitous services or any person from practising for the relief of any animal belonging to himself or herself. The Act does not apply to commissioned veterinary surgeons in the United States Army.

